# Integrative Transcriptome and Metabolome Analysis Reveals the Regulatory Mechanism Underlying the Potential Antioxidant Activity of Neohesperidin Dihydrochalcone-L-Arginine Complex in *Caenorhabditis elegans*

**DOI:** 10.3390/foods15071235

**Published:** 2026-04-04

**Authors:** Ping Chen, Siming Zhu, Menghan Tian, Yutao Wang, Liang Chen, Zhendong Wang

**Affiliations:** 1 Guangdong Province Key Laboratory for Green Processing of Natural Products and Product Safety, School of Food Science and Engineering, South China University of Technology, Guangzhou 510640, China; 2 Guangdong Pilot Platform of Modern Agriculture and Food for Lingnan Characteristic Fruits and Vegetables Bio-Processing (West Guangdong), Yangjiang Research Institute, South China University of Technology, Yangjiang 529500, China; 3 Green Processing Industry College for Lingnan Specialty Fruits and Vegetables, Yangjiang Tilapia Storage and Processing Engineering Technology Research Center, School of Food Science and Engineering, Guangdong Ocean University, Yangjiang Campus, Yangjiang 529500, China; 4 Key Laboratory of Biological Resources and Ecology of Pamirs Plateau of Xinjiang Uygur Autonomous Region, Kashi University, Kashi 844000, China; 5 Shandong Benyue Biological Technology Co., Ltd., Dongying 257000, China

**Keywords:** neohesperidin dihydrochalcone, oxidative stress, neohesperidin dihydrochalcone-L-arginine complex, *Caenorhabditis elegans*, antioxidant, transcriptomics, metabolomics

## Abstract

Neohesperidin dihydrochalcone (NHDC) has been confirmed to possess excellent nutritional activities as a natural flavonoid low-calorie sweetener, but its practical application in the food industry was greatly limited due to its low water solubility. The potential NHDC activity against oxidative stress (OS) diseases was explored through network pharmacology and molecular docking technology, and a highly water-soluble NHDC-L-arginine complex (NL) was prepared by combining NHDC with L-arginine to overcome this technical bottleneck. Meanwhile, the enhancement of antioxidant capacity markers under non-stressed conditions following NL treatment was systematically investigated in *Caenorhabditis elegans* (*C. elegans*), and transcriptomic and metabolomic analyses were integrated to reveal the potential regulatory mechanism at the molecular and metabolic levels. It was found that NHDC could exert potential anti-OS effects by targeting and binding to key proteins such as CYP19A1, TYR, EPHX2, TDP1, ESR1, and SLC5A1. In addition, the MDA level in *C. elegans* after NL intervention was significantly reduced to 0.65 ± 0.06 nmol/mg prot, while the activities of antioxidant enzymes T-SOD, GSH-Px, and CAT were significantly increased to 48.83 ± 1.75 U/mg prot, 112.95 ± 0.55 U/mg prot, and 6.30 ± 0.16 U/mg prot, respectively. Longevity regulating pathway–worm was identified as a potential key signaling pathway for NL to regulate the enhancement of antioxidant capacity markers under non-stressed conditions of *C. elegans* at the molecular level, and the pentose phosphate pathway was the core metabolic pathway. These results could offer theoretical support for the potential development of NHDC and NL in the field of antioxidants, as well as their large-scale applications in the functional food and flavored food industries.

## 1. Introduction

The incidence of diabetes caused by high-sugar and high-fat diets has increased exponentially in the past, with the total number of patients exceeding 500 million, and it is expected that the number of patients will continue to grow in the future [1]. Diabetic patients cannot effectively regulate blood glucose metabolism after ingesting sucrose, glucose, and other traditional sugars due to insufficient insulin secretion or insulin resistance, resulting in a sharp rise in blood glucose levels, which is harmful to health [2]. Therefore, the development of natural sweeteners with both enjoyable sweet tastes and a low glycemic index could meet the sensory needs of diabetic patients for sweet taste without causing significant fluctuations in blood sugar, which provides a critical pathway for diabetic patients to pursue sweet material enjoyment, supporting the transformation of the low sugar food industry [3]. Neohesperidin dihydrochalcone (NHDC), a chalcone natural sweetener, could be efficiently obtained by hydrogenation of its precursor neohesperidin (NH), and it was found that NHDC had the characteristics of being low in calories and high in sweet taste, and is used in the production of fruit juice, flavored dairy products and baked goods in the food industry [4]. In addition, NHDC, as a natural flavonoid compound, has been found to have significant effects in delaying kidney disease, preventing and treating food allergy, improving gut microbiota disorder, and alleviating liver injury symptoms, which could provide a basis for its application in the development of functional sweet foods [5,6,7].

The poor water solubility of NHDC limits the development and application of functional and flavor foods in the food industry, as it is difficult to disperse in aqueous solution systems and may cause precipitation, stratification, and other problems, affecting the sensory quality and stability of products [8]. Meanwhile, NHDC is faced with the disadvantages of low dissolution efficiency in the food processing industry, increased complexity and cost of the production process, and reduced potential physiological functions [9]. Therefore, it has become the key to achieving a breakthrough and expanding its application in the field of health food to improve the water solubility of NHDC through modification technology. Existing solubilization approaches for small flavonoid molecules mainly include chemical modification (such as glycosylation), physical modification (such as microencapsulation), and formulation with synthetic surfactants, but these methods often suffer from high production costs, complex operations, or loss of intrinsic functional properties of NHDC [10]. As a natural basic amino acid, L-arginine (L-arg) contains polar hydrophilic groups such as positively charged guanidine and amino groups, which can form stable complexes with poorly water-soluble flavonoids through intermolecular interactions such as hydrogen bonds, electrostatic interactions, and hydrophobic interactions, breaking the hydrophobic aggregation between small flavonoid molecules [11]. At the same time, it can introduce hydrophilic groups into the complex, improving the aqueous dispersibility of the whole molecule [12]. In the food industry, L-arg exists widely in natural animal and plant proteins, and the complexation between L-arg and small flavonoid molecules does not require complex equipment, overcoming the complexity and cost issues of existing physical/chemical modification methods [13]. In addition, L-arg is one of the essential amino acids for humans, which has physiological functions such as regulating body metabolism and improving immunity [14]. The complexation process of L-arg and small flavonoid molecules occurs via intermolecular non-covalent bonds, which do not change the key sites of the molecular structure and physiological activity of small flavonoid molecules, thus avoiding the functional loss associated with chemical modification or surfactant addition [15]. Therefore, the complex formed by the reaction of L-arg with NHDC can not only enhance water solubility but also retain the high sweetness and low-calorie characteristics of original NHDC, showing potential advantages in improving the nutritional value of products.

Although previous studies have reported co-amorphous systems of flavonoids with L-arginine exhibiting enhanced solubility and bioactivity, as well as antioxidant investigations in *C. elegans* combined with omics approaches, systematic investigations focusing on the nutritional properties, physiological activities, and underlying mechanisms of the specific NHDC–L-arginine complex (NL) remain limited [15,16]. In particular, few studies have directly compared NL with existing NHDC formulations or other reported flavonoid–arginine complexes in terms of composition, solubility, physical stability, and distinct biological endpoints, and the integrated regulatory cascade from network pharmacology and molecular docking to in vivo *C. elegans* assays coupled with multi-omics has not been established for this complex. Therefore, exploring the antioxidant and nutritional physiological characteristics of NL in vivo and analyzing its potential mechanism can provide theoretical support for the development of functional foods based on this complex, while offering a critical pathway for the modification and high-value application of natural sweeteners. As a classic model organism for evaluating antioxidant activity, *Caenorhabditis elegans (C. elegans*) has advantages such as a highly conserved oxidative stress regulatory pathway with mammals, a short life cycle with rapid reproduction, and high sensitivity to active substance intervention, which could efficiently achieve in vivo gradient verification of antioxidant activity, and the results have cross-species reference value, providing basis for subsequent mechanistic studies [16,17]. Meanwhile, transcriptomics and metabolomics could complement each other in terms of mRNA transcription and metabolite response, comprehensively analyzing the differentially expressed mRNA, core regulatory pathways, and characteristic metabolite changes after active substance intervention [18,19]. In addition, it can provide comprehensive and in-depth technical support for elucidating the antioxidant mechanism of active substances through correlation analysis among gene expression, metabolic regulation, and in vivo antioxidant biochemical indicators [20].

In this study, putative target proteins of NHDC involved in oxidative stress regulation were predicted *via* network pharmacology, and potential interactions with these targets were further assessed using molecular docking, providing preliminary theoretical support for the bioactivity of NL. At the same time, NL was prepared by complexation of NHDC with L-arg, and the potential antioxidant activity of NL was evaluated by investigating its effects on the antioxidant capacity of *C. elegans* under non-stressed conditions. The effects of NL intervention on the metabolites and mRNA transcription of *C. elegans* were profiled using transcriptomic and metabolomic approaches, and the putative mechanisms by which NL may enhance antioxidant capacity in *C. elegans* were explored at the metabolic and molecular levels through correlation analysis. The main novelty of this work lies not only in the development of NL with enhanced aqueous solubility, but also in the establishment of an integrated methodological pipeline combining network pharmacology, molecular docking, in vivo *C. elegans* evaluation, and multi-omics correlation to dissect its biological effects. These results could further reveal the nutritional value of NL related to its potential antioxidant activity on the basis of improved water solubility of NHDC, and provide technical support for its application as a natural sweetener in the development of functional and flavor foods.

## 2. Materials and Methods

### 2.1. Network Pharmacology Analysis of NHDC

The target proteins of NHDC were obtained through searching the Swiss Target Prediction database, and those with a score greater than 0 were considered as effective potential target proteins. “Oxidative stress” was used as a keyword to obtain disease target proteins in the GeneCards database, and those with a score greater than 1 were selected as effective disease target proteins. The overlapping target proteins between NHDC and oxidative stress diseases (OS) were obtained through preliminary analysis on the JVENN platform, and the key target proteins regulated by NHDC in OS were visualized through Cytoscape 3.8.0 software. Additionally, the obtained key target proteins were uploaded to the DAVID database, and potential pathways of NHDC intervention in OS were identified by mining GO and KEGG enrichment pathways analysis. The screening criteria were set at *p* < 0.05, and the top ten pathways ranked by gene ratio were considered as effective pathways. GO enrichment pathway analysis included biological process (BP), cellular component (CC), and molecular function (MF) analysis [16].

### 2.2. Molecular Docking Study

The 3D structural information of the key target proteins was obtained by searching the PDB database for the 13 key target proteins (SRD5A1, SLC5A4, CYP19A1, TYR, SLC5A1, SLC5A2, PTPN1, AKR1B1, TDP1, ESR1, PTPN2, CA14 and EPHX2) involved in the network pharmacology screening of NHDC-regulated OS. The target proteins were individually imported into AutoDock Tools 1.5.7 software for water removal and hydrogenation. The 2D structure of NHDC was obtained by searching the PubChem platform, and was subjected to energy minimization and hydrogen bond addition treatment using Avogadro 2.0.0. The processed NHDC small-molecule ligand and various macromolecular target proteins were separately imported into AutoDock Tools 1.5.7 software for molecular docking and analysis. The binding energies and hydrogen bond docking sites between the small-molecule ligand NHDC and different key target proteins were investigated as key indicators, and the results were visualized using PyMOL 2.1.0 to elucidate the potential mechanism of NHDC in regulating OS diseases [21].

### 2.3. Preparation of NL

NHDC was provided by Shandong Benyue Biological Technology Co., Ltd. (Dongying, China), and L-arginine was purchased from Shanghai Yuanye Bio-Technology Co., Ltd. (Shanghai, China). NHDC and L-arginine were mixed at a molar ratio of 1:3 and placed in a mortar. Anhydrous ethanol was continuously added dropwise until it accounted for approximately 20% of the total solid mass, and grinding was continued at room temperature for 1 h. During this process, anhydrous ethanol is continuously supplemented to the reaction system to maintain a slightly moist paste-like consistency, preventing incomplete reaction between NHDC and L-arg due to ethanol evaporation. NL was obtained by transferring the reaction product to a vacuum drying oven and drying under vacuum at 40 °C for 6 h to remove the residual solvent.

### 2.4. Analysis of Antioxidant Activity in C. elegans

*C. elegans* was used to evaluate the enhancement of antioxidant capacity markers by NL under non-stressed conditions, and its synchronization and OP50 amplification (the food of *C. elegans*) were conducted according to the method described by Lee et al. [22]. After synchronization of *C. elegans* was completed, it was randomly divided into 4 groups, including the non-intervention group (NFD), the low-concentration NL intervention group (NL mixed into OP50 to form a 50 μg/mL intervention, NL-L), the medium-concentration NL intervention group (NL mixed into OP50 to form a 100 μg/mL intervention, NL-M), the high-concentration NL intervention group (NL mixed into OP50 to form a 200 μg/mL intervention, NL-H), and the specific information of grouping and intervention is shown in Table 1 [20]. NL showed superior antioxidant activity over NHDC at a dose of 100 μg/mL in our prior work; 50 μg/mL and 200 μg/mL were selected to test the minimal effective dose and upper safe limit, respectively. Our previous studies confirmed 100 μg/mL as a safe dose with no adverse effects on *C. elegans* survival, locomotion, or food intake [20]. Different NL interventions were applied to *C. elegans*, and after 72 h of intervention, *C. elegans* were collected, lysed, homogenized, and centrifuged to obtain the supernatant. The protein concentration of supernatant was measured through a BCA kit (Beijing LABLEAD Trading Co., Ltd., Beijing, China), and antioxidant-related biochemical indicators were determined according to the kit method (Nanjing Jiancheng Biological Engineering Research Institute, Nanjing, China), including malondialdehyde (MDA), total superoxide dismutase (T-SOD), glutathione peroxidase (GSH-Px), and catalase (CAT) levels.

### 2.5. Transcriptomic Analysis

After 3 d of different NL interventions (0 or 200 μg/mL), *C. elegans* were collected, and they were repeatedly washed three times with M9 buffer to remove the bacterial solution on their surface. Total RNA was extracted, and its concentration and purity were tested to ensure that the sample concentration was above 30 ng/mL; the OD260/280 ratio was between 1.8 and 2.2, which met the requirements for library construction. The library was obtained by reverse transcribing the processed mRNA fragments into cDNA, purifying and sorting the fragments, and performing PCR amplification, and the amplified products were sequenced on the NovaSeq X Plus platform. The sequencing results were aligned with the reference genome of *C. elegans* to obtain mRNA transcription information and screen for differentially expressed mRNAs, and the screening criteria were FDR < 0.05 and |log2FC| ≥ 1. The characteristic mRNAs were visualized through sample correlation analysis, PCA, and volcano plot analysis of the results. The GO enrichment pathways of characteristic mRNAs, including BP, CC, and MF, were obtained by utilizing Goatools 1.6.4. Meanwhile, KEGG pathway enrichment analysis was conducted on characteristic mRNAs through the Python (3.2.2) scipy package to identify the key signaling pathways involved in the enhancement of antioxidant capacity of *C. elegans* induced by NL intervention. Key mRNAs involved in the critical signaling pathways were screened based on transcriptomic results, and their relative expression levels were presented. In addition, the correlations between characteristic mRNA and the enhancement of antioxidant capacity markers under non-stressed conditions of *C. elegans* (MDA, T-SOD, GSH-Px and CAT) were investigated through Spearman correlation analysis to explore the potential association of NL intervention with the enhancement of antioxidant capacity at the molecular expression level [23].

### 2.6. Metabolomics Analysis

*C. elegans* were collected after 3 d of different NL interventions (0 or 200 μg/mL), and the bacterial solution on the surface of *C. elegans* was removed by repeatedly washing with M9 buffer three times. The samples were homogenized and lysed on ice, and the supernatant containing metabolites was obtained after centrifugation, transferred to a sample vial for instrumental detection. After the results were downloaded, they were imported into Progenesis QI 2.0 (Waters Corporation, Milford, MA, USA) software for matching and qualitative analysis of metabolite information by comparing with the HMDB database and Metlin database. The characteristic metabolites were obtained by calculating the Variable Importance in Projection (VIP) values and *p*-values of metabolites in *C. elegans* of the NL-H group and NFD, with screening criteria of VIP > 1 and *p* < 0.05. The macroscopic changes in the antioxidant capacity enhancement of *C. elegans* by NL intervention were visualized through PCA and PLS-DA analysis, as well as volcano plots, and the characteristic metabolic pathways were identified by matching with the KEGG database. Additionally, the correlations between characteristic metabolites and the enhancement of antioxidant capacity markers under non-stressed conditions (MDA, T-SOD, GSH-Px and CAT) were investigated in *C. elegans* under NL intervention were analyzed through Spearman correlation analysis to explore potential relationships related to the enhancement of antioxidant capacity under non-stressed conditions in *C. elegans* under NL intervention at the metabolite level [24].

### 2.7. Statistical Analysis

Data analysis and figure plotting were completed by IBM SPSS Statistics 26 software and Origin 2022 software, respectively. All experimental results were repeated at least three times and were presented as mean ± SD value. The differences in antioxidant-related biochemical indicators between the NFD and the groups of *C. elegans* treated with NL of different concentrations were tested through one-way ANOVA followed by Duncan multiple comparison. The correlation among the enhancement of antioxidant capacity under non-stressed conditions, expression of characteristic mRNA, and characteristic metabolites in *C. elegans* treated with high-concentration NL was analyzed through the Spearman method.

## 3. Results

### 3.1. Network Pharmacology Analysis of NHDC in the Regulation of OS Diseases

**Figure 1:**
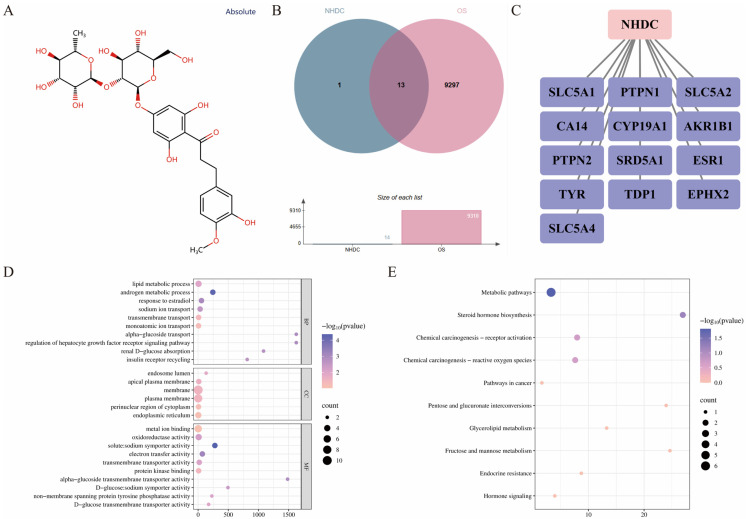
Network pharmacology analysis of neohesperidin dihydrochalcone (NHDC) in intervening oxidative stress (OS) diseases. (**A**): Chemical structure of NHDC. (**B**): Venn diagram showing the overlap between NHDC-related targets and OS-related targets. The 13 overlapping targets were identified as genes shared between the predicted target set of NHDC and the disease target set of OS. (**C**): Network diagram illustrating the interactions between NHDC and its 13 overlapping targets with OS. (**D**): GO enrichment analysis of overlapping targets (BP: biological process; CC: cellular component; and MF: molecular function). (**E**): KEGG pathway enrichment analysis of overlapping targets.

NHDC has exhibited a variety of health-regulatory activities, and it was confirmed that OS was potentially associated with dyslipidemia, gut microbiota imbalance, diabetes, inflammation and other diseases [25,26]. Therefore, it could provide a theoretical basis and research potential for the development of functional foods centered on OS with potential multiple health-regulatory activities through exploring and mining the potential target proteins of NHDC in regulating OS-related diseases by network pharmacology (Figure 1). In total, 13 core overlapping targets were identified between the 14 targets of NHDC and the 9310 targets of OS-related diseases, namely SLC5A1, PTPN1, SLC5A2, CA14, CYP19A1, AKR1B1, PTPN2, SRD5A1, ESR1, TYR, TDP1, EPHX2 and SLC5A4, which suggested that NHDC might exert a potential critical ameliorative effect on the regulation of OS-related diseases (Figure 1A–C). In addition to exerting potential anti-OS effects, these overlapping target proteins also exhibited potential regulatory roles in ameliorating various health conditions, including tumor growth inhibition and drug-induced nephropathy [27,28]. AKR1B1 has been demonstrated to play a potential role in attenuation of cell proliferation, invasion and migration in ovarian cancer cells [27]. Targeted regulation of CYP19A1 could ameliorate pathological states of oxidative stress and hormonal imbalance in rats with letrozole-induced polycystic ovary syndrome [29]. Moreover, the improvement of interstitial pneumonia has been found to be closely associated with the targeted modulation of ESR1 [30]. The multiple nutritional benefits exerted by the targeted regulation of these proteins could provide potential significant references for in-depth exploration of the multi-dimensional nutritional activities of NHDC, broadening the application prospects of NHDC as a potential functional sweetener in the food industry.

The overlapping target proteins were uploaded to the DAVID database to explore their potential signaling pathways in regulating OS (Figure 1D,E). In the GO functional enrichment analysis, NHDC mainly participated in BP-related pathways, including the lipid metabolic process, androgen metabolic process, and response to estradiol; the major enriched pathways were endosome lumen, apical plasma membrane and membrane in terms of CC; the key involved pathways were metal ion binding, oxidoreductase activity and solute:sodium symporter activity for MF (Figure 1D). KEGG pathway enrichment analysis indicated that NHDC exerted its OS-regulatory effect mainly by participating in metabolic pathways, steroid hormone biosynthesis, and chemical carcinogenesis-receptor activation (Figure 1E). Enrichment analyses of GO functions and KEGG pathways provided an important reference for exploring the potential mechanisms of NHDC in intervening in OS-related diseases. Among them, the lipid metabolic process identified by GO enrichment offered critical molecular evidence for its activity in regulating diseases associated with lipid metabolism disorders beyond anti-OS [31]. The significant enrichment of oxidoreductase activity confirmed the prominent anti-oxidative stress effect of NHDC, implying that it could directly participate in the scavenging of reactive oxygen species and the maintenance of redox homeostasis in organisms by regulating this core molecular function [32]. Meanwhile, the benign regulation of the steroid hormone biosynthesis pathway has been proven to exert a significant effect on alleviating lung injury, restoring blood–gas barrier function, and reducing serum inflammatory factor levels in sepsis-associated mice [33]. These GO and KEGG enrichment results predictively elucidated the core molecular mechanisms underlying the potential anti-OS activity of NHDC, and suggested its potential in regulating lipid metabolism disorders and alleviating inflammation-related pathological damage, laying a molecular foundation for further exploring the multi-target and multi-functional regulatory effects of NHDC in the intervention of OS-associated diseases.

### 3.2. Mining of Protein Target Information for NHDC in the Regulation of OS Diseases

**Figure 2:**
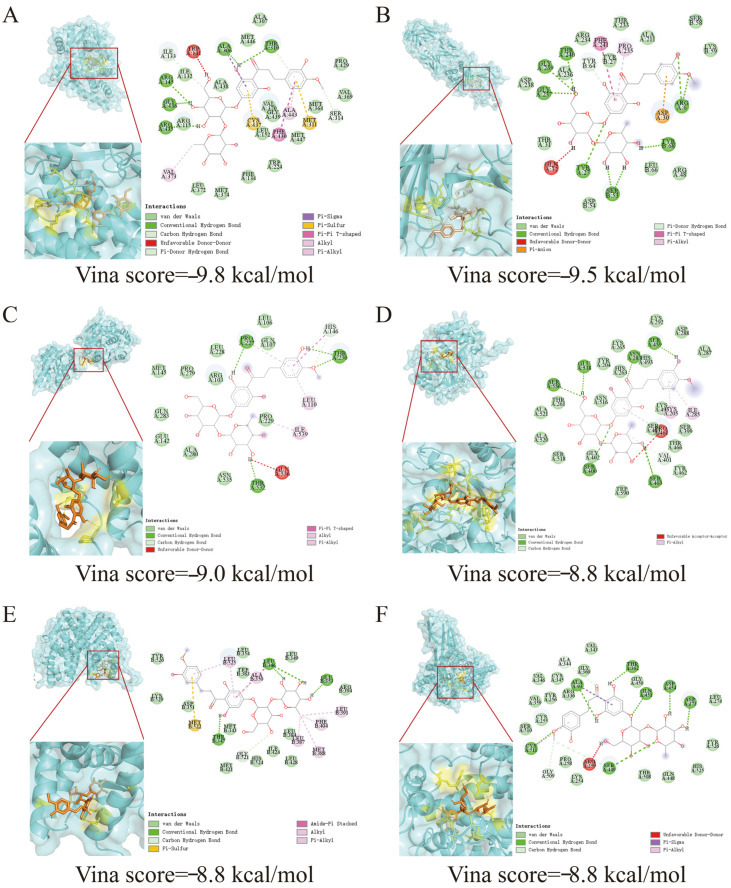
Molecular docking results of NHDC in regulating OS diseases. (**A**) The molecular docking diagram of NHDC and protein CYP19A1; (**B**) The molecular docking diagram of NHDC and protein TYR; (**C**) The molecular docking diagram of NHDC and protein EPHX2; (**D**) The molecular docking diagram of NHDC and protein TDP1; (**E**) The molecular docking diagram of NHDC and protein ESR1; (**F**) The molecular docking diagram of NHDC and protein SLC5A1.

Molecular docking technology was employed to investigate the binding energy and docking site information of the core target proteins of NHDC in regulating OS diseases, which were screened by network pharmacology, clarifying the potential mechanism underlying NHDC-mediated intervention in OS diseases, providing a theoretical reference for the research and development of relevant functional foods (Figure 2). In the docking study of NHDC with OS disease target proteins, CYP19A1 ranked the first with a binding energy of −9.8 kcal/mol, followed by TYR with −9.5 kcal/mol and EPHX2 with −9.0 kcal/mol, while TDP1, ESR1 and SLC5A1 exhibited an identical binding energy of −8.8 kcal/mol, which were the top six target proteins identified (Figure 2, Table 2). In molecular docking studies, the lower the binding energy, the more energy is released when NHDC binds to the target protein, making the complex formed by them more difficult to dissociate and enabling the ligand to bind to and regulate the function of the target protein with higher efficiency [34,35]. These results indicated stronger binding affinity and more stable binding interactions between NHDC and the target proteins, which demonstrated that NHDC could achieve highly efficient and stable specific binding to target proteins, exerting its potential anti-OS activity through the targeted regulation of the biological functions of target proteins.

In the binding site analysis, the docking interactions between NHDC and target proteins were identified to mainly involve van der Waals forces, conventional hydrogen bonds and C-H hydrogen bonds. Among them, the hydrogen bonding sites of CYP19A1 with NHDC were mainly ALAA306, THRA310, ARGA145, GLYA436 and ARGA435, the hydrogen bonding sites of TYR with NHDC included THRA240, GLYA239, GLYA237, ARGA6, TYRB68, SERB53 and TYRA27, as well as the hydrogen bonding sites of TYR with NHDC included PROA227, THRA230 and THRA532 (Figure 2, Table 2). These hydrogen bonding sites and their corresponding target proteins jointly form the molecular binding basis for NHDC to exert its regulatory effect on OS, which provides structural support for the stable binding of NHDC to target proteins and the subsequent regulation of biological activities (Figure 2 and Table 2). The result mining of the binding sites between NHDC and target proteins clarified the specific molecular interaction modes and key binding residues between NHDC and OS-related target proteins, highlighting the critical role of hydrogen bonds in stabilizing the ligand-protein complex [36]. Meanwhile, the structural specificity and diversity of the binding sites could further explain the multi-target regulatory characteristics of NHDC in potential OS modulation, providing structural support for its stable binding to target proteins and subsequent regulation of biological activities, as well as a theoretical basis for the structural modification and functional optimization of NHDC in the potential development of OS-targeted functional foods.

### 3.3. Effects of NL Intervention on Antioxidant Capacity in C. elegans

**Figure 3:**
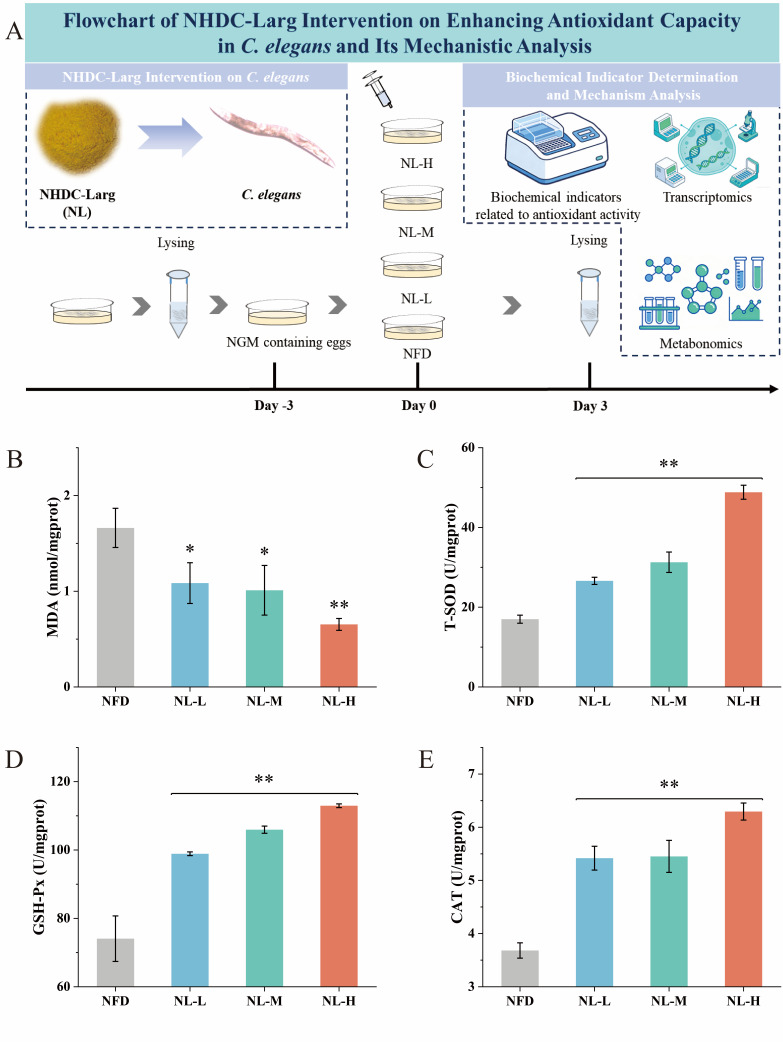
Effects of NHDC-Larg (NL) intervention on the antioxidant capacity of *C. elegans*. (**A**): Schematic diagram of the experimental design and treatment workflow. (**B**): Malondialdehyde (MDA) levels in *C. elegans* following NL intervention. (**C**): Total superoxide dismutase (T-SOD) activity in *C. elegans* after NL treatment. (**D**): Glutathione peroxidase (GSH-Px) activity in *C. elegans* under NL intervention. (**E**): Catalase (CAT) activity in *C. elegans* treated with NL. Data are presented as mean ± standard deviation (SD); n = 3 independent biological replicates. Statistical analysis was performed using one-way analysis of variance (ANOVA) followed by Tukey’s post hoc test. Asterisks indicate significant differences compared with the NFD: * *p* < 0.05, ** *p* < 0.01.

*C. elegans* was used as a biological model to evaluate the enhancement of antioxidant capacity markers under non-stressed conditions of NL, and it was found that the levels of MDA, T-SOD, GSH-Px and CAT in *C. elegans* of the NFD were 1.66 ± 0.20 nmol/mg prot, 17.00 ± 1.01 U/mg prot, 74.07 ± 6.66 U/mg prot and 3.68 ± 0.14 U/mg prot, respectively (Figure 3). Compared with the NFD, the NL intervention significantly enhanced the enhancement of antioxidant capacity markers under non-stressed conditions of *C. elegans*. The levels of MDA, T-SOD, GSH-Px and CAT in *C. elegans* of the NL-L were 1.09 ± 0.21 nmol/mg prot, 26.60 ± 0.90 U/mg prot, 98.90 ± 0.54 U/mg prot and 5.42 ± 0.22 U/mg prot, respectively; those in the NL-M group were 1.01 ± 0.26 nmol/mg prot, 31.27 ± 2.56 U/mg prot, 105.95 ± 1.05 U/mg prot and 5.45 ± 0.30 U/mg prot, respectively; and those in the NL-H group were 0.65 ± 0.06 nmol/mg prot, 48.83 ± 1.75 U/mg prot, 112.95 ± 0.55 U/mg prot and 6.30 ± 0.16 U/mg prot, respectively (Figure 3). The results of the enhancement of antioxidant capacity markers under non-stressed conditions in *C. elegans* treated with NL further validated the findings from NHDC network pharmacology and molecular docking studies, confirming the antioxidant potential of NL. Among the antioxidant-related biochemical indicators, MDA was a key product of lipid peroxidation induced by oxidative stress, and the reduced MDA level directly indicated that NL could effectively inhibit oxidative stress-mediated cellular damage [37,38]. Meanwhile, the synchronous enhancement of antioxidant enzyme activities demonstrated that NL could comprehensively activate the endogenous antioxidant defense system of *C. elegans* rather than regulating a single antioxidant pathway [39]. These results confirmed that NHDC possessed potential nutritional advantages as a flavonoid compound apart from serving as a low-calorie sweetener [40,41]. Notably, the oxidative stress response pathways and core antioxidant signaling mechanisms have been documented to exhibit relatively high conservation between *C. elegans* and mammals, which supported the reference value of the present findings for studies in higher organisms [42]. Moreover, the consistent improvement of multiple antioxidant indices in vivo suggested that NL might have comparable beneficial effects in mammalian systems and shows potential for future development as an antioxidant functional food ingredient [42]. However, *C. elegans*, as a lower organism model, could not fully represent the equivalent effects of NL in mammals. Therefore, subsequent studies should further explore NL multi-dimensional nutritional activities centered on antioxidant capacity using higher mammalian models such as mice.

### 3.4. Effects of NL Intervention on mRNA Transcription in C. elegans

**Figure 4:**
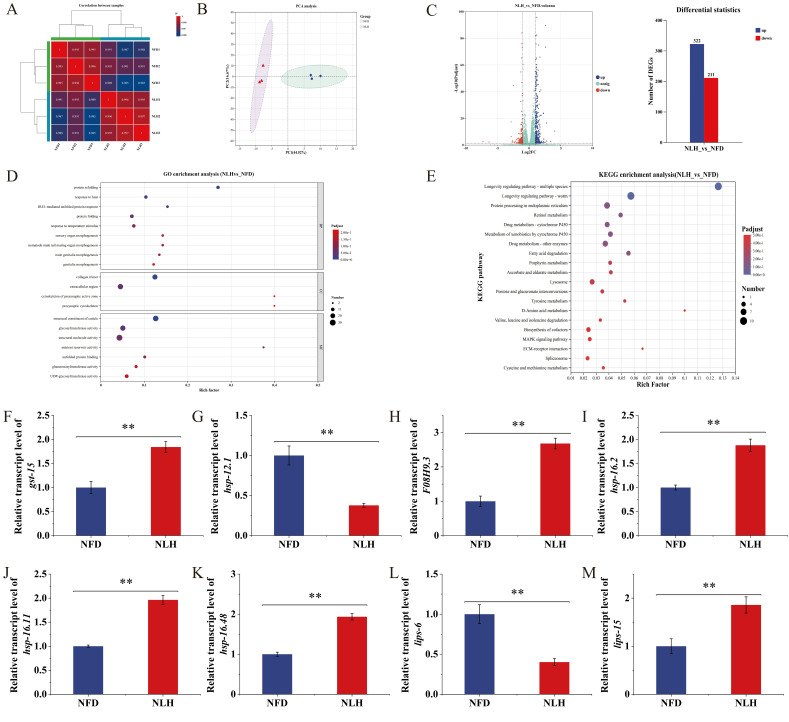
Effects of NL intervention on mRNA expression profiles in *C. elegans*. (**A**): Correlation analysis of mRNA expression data. (**B**): Principal component analysis (PCA) plot of mRNA expression patterns. (**C**): Volcano plot and statistical analysis for the identification of differentially expressed mRNAs. (**D**): Gene ontology (GO) pathway enrichment analysis of differentially expressed mRNAs in NL-treated *C. elegans*. (**E**): Kyoto Encyclopedia of Genes and Genomes (KEGG) pathway enrichment analysis of differentially expressed mRNAs in NL-treated *C. elegans*. (**F**–**M**): Relative transcription levels of representative differentially expressed mRNAs in NL-treated *C. elegans*. Data are shown as mean ± SD from n = 3 independent biological replicates. Statistical significance was determined by one-way ANOVA followed by Tukey’s post hoc test. Asterisks indicate significant differences compared with the NFD: ** *p* < 0.01.

The enhancement of antioxidant capacity markers under non-stressed conditions of *C. elegans* upon treatment with NL has been functionally linked to substantial alterations in mRNA expression, and transcriptomic profiling was applied to dissect the impact of NLH on the mRNA landscape of *C. elegans*, with the objective of identifying the molecular pathways underlying NLH-mediated antioxidant enhancement (Figure 4). High intra-group reproducibility was observed within both the NFD control group and the NLH intervention group, while a marked divergence in transcriptomic profiles was evident between the two groups; PCA results validated the significant transcriptomic separation between the NFD and NLH groups, corroborating the robustness of these results (Figure 4A,B).

NLH intervention resulted in the significant upregulation of 322 mRNA transcripts and the significant downregulation of 211 mRNA transcripts in *C. elegans* (Figure 4C). GO enrichment analysis was performed on these differentially expressed characteristic mRNAs, which revealed that the signaling pathways involved in BP mainly included protein refolding, response to heat, and IRE1-mediated unfolded protein response, those involved in CC mainly included collagen trimer, extracellular region, and cytoskeleton of presynaptic active zone, and those involved in MF mainly included structural constituent of cuticle, glycosyltransferase activity, and structural molecule activity (Figure 4D). Meanwhile, longevity regulating pathway–worm was identified as the key signaling pathway of NLH in regulating *C. elegans* through KEGG enrichment analysis, which was consistent with those of Miao et al. in their study on the transcriptional mechanism of olive fruit intervention in enhancing the antioxidant capacity of *C. elegans* (Figure 4E) [43]. Meanwhile, longevity regulating pathway–worm has been demonstrated to play a regulatory role in flavonoids improving hydrogenase regulation, lifespan modulation, and lipid metabolism, promoting the reproduction of *C. elegans* [44]. In the longevity regulating pathway–worm pathway, the key mRNAs gst-15, F08H9.3, hsp-16.48, hsp-16.11, hsp-16.2, and lips-15 were significantly up-regulated by 0.84-, 1.68-, 0.93-, 0.96-, 0.88-, and 0.86-fold, respectively, while mRNAs hsp-12.1 and lips-6 were significantly down-regulated by 62.23% and 59.81%, respectively (Figure 4F–M). Genes of the hsp family, especially the small heat shock protein hsp-16 subfamily, were core factors regulating stress resistance and lifespan in *C. elegans*, and the upregulation indicated that NL could enhance cell ability to repair oxidative damage by activating the heat shock response, while possessing the potential to synergistically extend lifespan [45]. As a member of the glutathione S-transferase family, the upregulation of gst-15 verified the antioxidant mechanism of NL in scavenging reactive oxygen species through strengthening detoxification pathways [46]. In contrast, the differential expression of the lips gene family suggested that NL might indirectly participate in the synergistic regulation of longevity and antioxidant capacity by modulating the balance of lipid metabolism [47].

### 3.5. Analysis of the Potential mRNA Transcriptional Mechanisms Underlying NL-Mediated Regulation of Antioxidant Capacity in C. elegans

**Figure 5:**
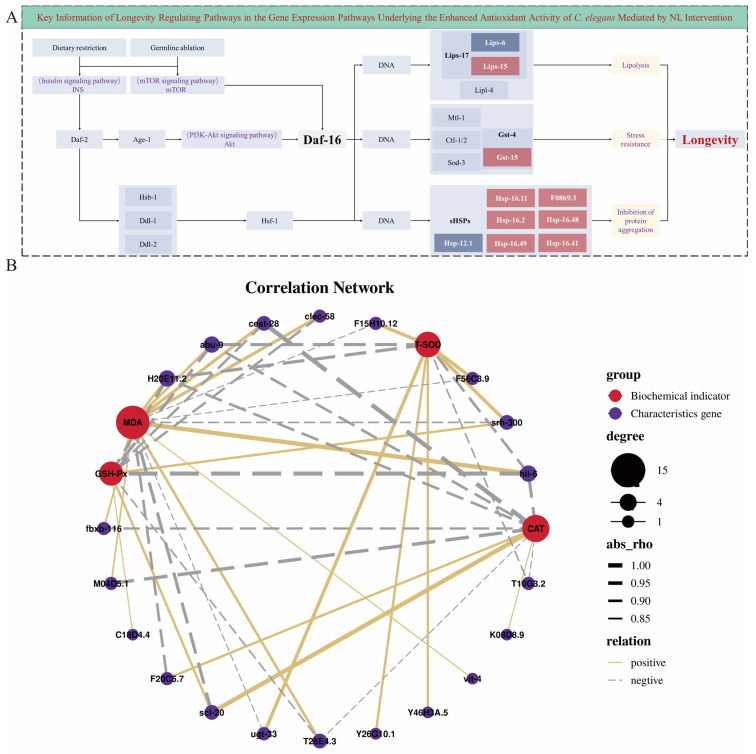
Mechanism analysis of NL-enhanced antioxidant capacity in *C. elegans*. (**A**): Key information of longevity regulating pathways in the mRNA expression pathways underlying the enhanced antioxidant activity of *C. elegans* mediated by NL intervention (Significantly up-regulated genes were marked in red; significantly down-regulated genes were marked in blue). (**B**): Correlation analysis between antioxidant-related biochemical indices and characteristic mRNAs in *C. elegans* treated with NL.

The core molecular pathways through which NL intervention enhanced the antioxidant capacity under non-stressed conditions of *C. elegans* by longevity regulating pathway–worm (Figure 5A). NL intervention acted on the daf-2 receptor in the INS pathway by mimicking dietary restriction, modulating the activities of age-1 and Akt, which enabled the core transcription factor daf-16 to translocate into the nucleus (Figure 5A) [48]. Meanwhile, daf-2 signaling activated the hsf-1 heat shock factor, driving alterations in the expression of downstream functional genes; the lipolysis-related gene lips-15 was significantly up-regulated in the lipid metabolism module, while lip-6 was down-regulated, which cooperatively promoted lipolysis (Figure 5A). Gst-15 was activated to enhance the stress resistance of *C. elegans* in the antioxidant and stress resistance module, and multiple small heat shock protein genes, including hsp-16.11, hsp-16.2, and F08h9.3, were significantly up-regulated in the protein homeostasis module, whereas hsp-12.1 was significantly down-regulated, collectively inhibiting protein aggregation (Figure 5A) [49,50]. These results suggested that NL intervention could enhance the antioxidant capacity under non-stressed conditions of *C. elegans* by orchestrating the coordinated activity of these functional modules, which collectively reinforced lipolysis, stress resistance and protein homeostasis (Figure 5A) [51].

The correlations between the enhancement of antioxidant capacity markers under non-stressed conditions and the expression of characteristic mRNAs in *C. elegans* treated with NL had explored, and the results revealed that the MDA level was significantly positively correlated with mRNAs hil-6, clec-58, cest-28, abu-9, fbxb-116, M04D5.1 and vit-4, and significantly negatively correlated with mRNAs scl-20 srh-300, and ugt-33; T-SOD level exhibited a significant positive correlation with mRNAs srh-300 and ugt-33, and a significant negative correlation with mRNAs abu-9 and hil-6 (Figure 5B). Additionally, GSH-Px level was significantly positively correlated with mRNAs scl-20 and srh-300, and significantly negatively correlated with mRNAs hil-6, abu-9, clec-58 and cest-28, while CAT level showed the same correlation pattern, with a significant positive correlation with mRNA scl-20 and a significant negative correlation with mRNAs cest-28, hil-6, abu-9 and fbxb-116 (Figure 5B). These results established the specific correlations between antioxidant-related biochemical indices and characteristic mRNAs in *C. elegans* under NL intervention, providing evidence for clarifying the regulatory link between gene expression and antioxidant biochemical phenotypes, as well as deepening the quantitative understanding of the molecular mechanism underlying NL antioxidant effects. Furthermore, the changes in antioxidant-related enzyme activities (T-SOD, GSH-Px, and CAT) and in MDA content were highly consistent with the transcriptional upregulation of corresponding antioxidant genes and key regulators in the longevity regulating pathway [52]. The elevated transcript levels of genes encoding antioxidant enzymes provided a molecular basis for the enhanced enzymatic activities observed, while the downregulation of oxidative damage-related processes at the transcriptional level supported the reduced MDA accumulation [23]. Such coordinated changes between gene expression profiles and biochemical phenotypes strongly suggest that NL enhances antioxidant capacity by systematically regulating the transcription of antioxidant-related genes, thereby improving redox homeostasis and reducing lipid peroxidation at the phenotypic level [53]. However, the correlations between antioxidant biochemical indices and mRNAs, without verifying their causal relationships, were revealed [54]. Therefore, subsequent studies need to confirm the regulatory effects of core mRNAs on oxidative biochemical indices through gene editing and phenotypic validation, integrate multi-dimensional omics technology to construct a complete regulatory network, and verify its conservation and application potential in higher organism models.

### 3.6. Effects of NL Intervention on Metabolites in C. elegans

**Figure 6:**
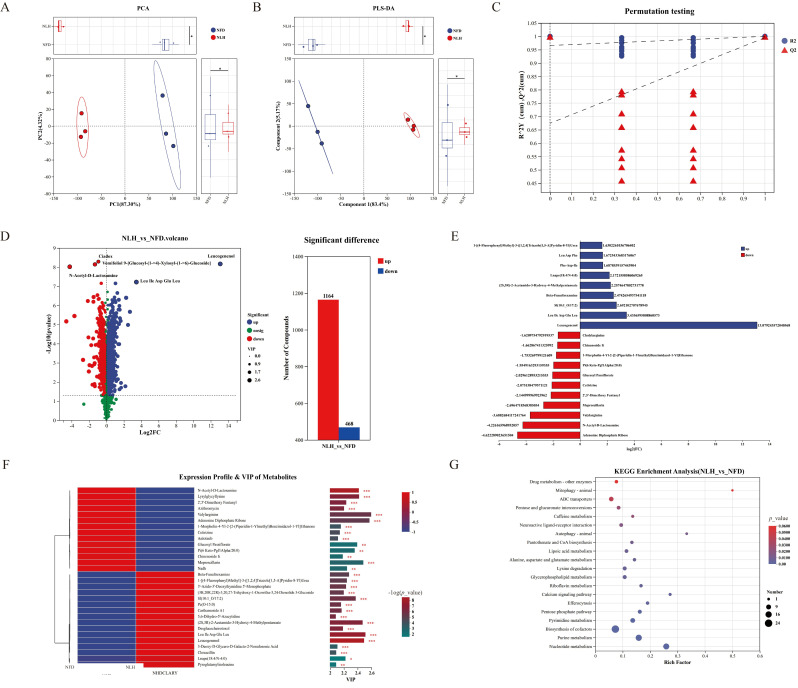
Effects of NL intervention on metabolites in *C. elegans*. (**A**): PCA plot. (**B**): PLS-DA plot. (**C**): Permutation testing. (**D**): Volcano plot and statistical chart of characteristic metabolite screening. (**E**): Characteristic metabolites screened by log2FC values. (**F**): Characteristic metabolites screened by VIP values. (**G**): KEGG pathway enrichment analysis of characteristic metabolites in *C. elegans* treated with NL. n = 3 independent biological replicates. Asterisks indicate significant differences compared with the NFD: * *p* < 0.05, ** *p* < 0.01, *** *p* < 0.001.

The bidirectional modulation of metabolites was demonstrated to exert a central regulatory role in the marked enhancement of antioxidant capacity under non-stressed conditions in *C. elegans* mediated by bioactive substances, and it was found that metabolites in *C. elegans* treated with NL were significantly changed, while permutation testing results verified the reliability of these metabolomic findings (Figure 6A–C). Compared with the NFD, 1164 metabolites were significantly up-regulated, and 468 metabolites were significantly down-regulated in *C. elegans* of the NLH group (Figure 6D).

Based on the screening of log_2_FC values, the significantly up-regulated metabolites mainly included leucogenenol, leu ile asp glu leu, beta-funaltrexamine, (2s,3r)-2-acetamido-3-hydroxy-4-methylpentanoate, phe-asp-ile, leu asp phe, and 1-[(4-fluorophenyl)methyl]-3-([1,2,4]triazolo [1,5-a]pyridin-8-yl)urea, while the significantly down-regulated metabolites mainly included adenosine diphosphate ribose, n-acetyl-d-lactosamine, valylarginine, meproscillarin, 2′,5′-dimethoxy fentanyl, cetirizine, glucosyl passiflorate, 1-morpholin-4-yl-2-[2-(piperidin-1-ylmethyl)benzimidazol-1-yl]ethanone, chinenoside ii, and cholylarginine (Figure 6E). Based on the screening of VIP values, the significantly up-regulated metabolites mainly included leu ile asp glu leu, leucogenenol, (2s,3r)-2-acetamido-3-hydroxy-4-methylpentanoate, beta-funaltrexamine, 1-[(4-fluorophenyl)methyl]-3-([1,2,4]triazolo[1,5-a]pyridin-8-yl)urea, 3′-azido-3′-deoxythymidine 5′-monophosphate, (3b,20r,22r)-3,20,27-trihydroxy-1-oxowitha-5,24-dienolide 3-glucoside, desglucocheirotoxol, carthamoside a1, 3-deoxy-d-glycero-d-galacto-2-nonulosonic acid, cloxacillin, pyroglutamylisoleucine, and 5,6-dihydro-5′-azacytidine, while the significantly down-regulated metabolites mainly included valylarginine, adenosine diphosphate ribose, meproscillarin, lysylglycyllysine, n-acetyl-d-lactosamine, glucosyl passiflorate, 2′,5′-dimethoxy fentanyl, azithromycin, chinenoside ii, 1-morpholin-4-yl-2-[2-(piperidin-1-ylmethyl)benzimidazol-1-yl]ethanone, cetirizine, and anlotinib (Figure 6F).

Potential metabolic pathways were obtained through KEGG pathway enrichment analysis of the acquired characteristic metabolites, and the top five potential metabolic pathways were ABC transporters, lysine degradation, pentose phosphate pathway, glycerophospholipid metabolism, and neuroactive ligand–receptor interaction according to the number of metabolites involved in each pathway (Figure 6G). According to the enrichment factor ranking, the top five potential metabolic pathways were autophagy–animal, calcium signaling pathway, efferocytosis, riboflavin metabolism, and pentose phosphate pathway (Figure 6G). Additionally, based on statistical significance (*p*-value), the top five potential metabolic pathways were identified as pentose phosphate pathway, efferocytosis, calcium signaling pathway, riboflavin metabolism and lysine degradation (Figure 6G). Notably, the pentose phosphate pathway was confirmed as the key metabolic pathway enriched in metabolite regulation of *C. elegans* in the NLH group, as it was consistently present among the top-ranked pathways across all three screening methods (Figure 6G). The pentose phosphate pathway has been confirmed as a core pathway regulating oxidative stress resistance and aging in *C. elegans*, which could provide reducing power for glutathione synthesis by producing NADPH, and the generated ribose-5-phosphate participates in nucleic acid metabolism, directly influencing cellular oxidative damage repair and lifespan maintenance [55,56,57]. These results clarified that the pentose phosphate pathway could serve as a key hub linking oxidative stress and aging regulated by NL, laying a foundation for elucidating the mechanism underlying the enhancement of antioxidant capacity under non-stressed conditions in *C. elegans* upon NL intervention.

### 3.7. Analysis of the Potential Metabolomic Mechanisms Underlying NL-Mediated Regulation of Antioxidant Capacity in C. elegans

**Figure 7:**
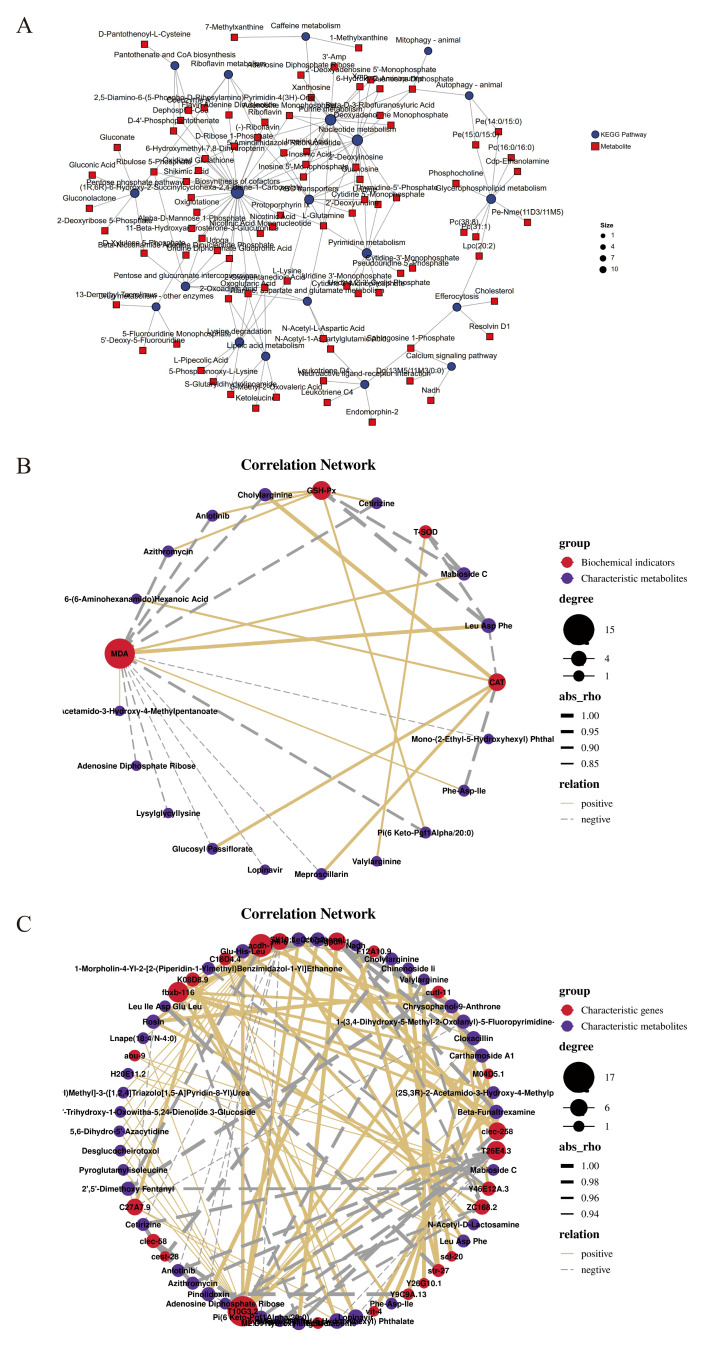
Mechanism analysis of NL-enhanced antioxidant capacity in *C. elegans* through the mRNA transcription and metabolites. (**A**): Network diagram. (**B**): Correlation analysis between antioxidant-related biochemical indices and characteristic metabolites in *C. elegans* treated with NL. (**C**): Correlation analysis between characteristic mRNAs and characteristic metabolites in *C. elegans* treated with NL.

It could elucidate the metabolic regulatory mechanisms underlying the enhanced antioxidant capacity under non-stressed conditions of *C. elegans* treated with NL at the metabolite level through exploring the metabolite–KEGG pathway association network, and provide precise metabolic network evidence for revealing how NL enhanced antioxidant capacity under non-stressed conditions and screening critical biomarkers by mapping the associations between core pathways and key metabolites (Figure 7A). Among these pathways, the pentose phosphate pathway served as one of the core nodes, directly connecting to key metabolites such as d-ribose 5-phosphate and 6-phosphogluconate through multiple links (Figure 7A) [58]. Meanwhile, it could interact with core pathways like biosynthesis of cofactors, and form a complex regulatory network with pathways such as purine metabolism and pyrimidine metabolism through metabolite sharing (Figure 7A) [59]. These results confirmed the pivotal role of the pentose phosphate pathway in maintaining cellular redox balance and providing precursors for nucleotide synthesis, and reflected its critical position in the metabolic network [60].

It could reveal the potential metabolic mechanisms underlying physiological function changes from the association between macroscopic physiological phenotypes and microscopic metabolic molecules through mining the correlations between biochemical indices and characteristic metabolites in *C. elegans* under NL intervention, providing critical correlative evidence for the precise elucidation of the biological processes by which metabolites exerted antioxidant capacity under non-stressed conditions in *C. elegans* under NL intervention and for the screening of potential biomarkers (Figure 7B). MDA was identified to exhibit a significant positive correlation with metabolites leu asp phe, mabioside c, phe-asp-ile, and (2s,3r)-2-acetamido-3-hydroxy-4-methylpentanoate, as well as a significant negative correlation with metabolites cetirizine, cholylarginine, anlotinib, azithromycin, adenosine diphosphate ribose, lysylglycyllysine, meproscillarin, glucosyl passiflorate, mono-(2-ethyl-5-hydroxyhexyl) phthalate, and lopinavir (Figure 7B). T-SOD showed a significant positive correlation with metabolite valylarginine, and a significant negative correlation with metabolites mabioside c and leu asp phe (Figure 7B). GSH-Px was significantly positively correlated with metabolites cetirizine, cholylarginine, anlotinib, and azithromycin, and significantly negatively correlated with metabolites leu asp phe and mabioside c (Figure 7B). CAT presented a significant positive correlation with metabolites cholylarginine, meproscillarin, glucosyl passiflorate, and 6-(6-aminohexanamido) hexanoic acid, and a significant negative correlation with metabolites phe-asp-ile and leu asp phe (Figure 7B). The association between macroscopic antioxidant physiological phenotypes and microscopic gene expression profiles has been established by analyzing the correlations between biochemical indices and differentially expressed mRNAs in *C. elegans* under NL intervention, providing important correlative evidence for elucidating the molecular processes by which key genes mediate the enhancement of antioxidant capacity markers under non-stressed conditions and for identifying core regulatory genes [24]. However, these results only demonstrated the correlations between antioxidant biochemical indices and transcript levels without confirming their causal relationships [61]. Therefore, further studies using gene overexpression, knockout, or RNAi combined with phenotypic validation are required to verify the regulatory roles of hub genes in modulating oxidative stress-related biochemical indices.

### 3.8. Correlation Analysis of Characteristic mRNA and Characteristic Metabolite Levels Underlying NL-Mediated Enhancement of Antioxidant Capacity in C. elegans

It could enable the systematic elucidation of the molecular pathways underlying the conversion of gene expression regulation into metabolic phenotypes during the enhancement of antioxidant capacity in *C. elegans* upon NL intervention from the synergistic perspective of transcriptional regulation and metabolic response through deciphering the intrinsic correlation between the characteristic mRNA expression profiles and characteristic metabolite profiles, providing critical multi-omics integrated evidence and core theoretical support for the comprehensive interpretation of the molecular regulatory mechanisms governing its antioxidant effects. It was found that mRNA T26E4.3 was significantly negatively correlated with metabolites adenosine diphosphate ribose, cetirizine, anlotinib, azithromycin, lysylglycyllysine, mono-(2-ethyl-5-hydroxyhexyl) phthalate and lopinavir, while mRNA gpdh-1 was significantly negatively correlated with metabolites leucogenenol, beta-funaltrexamine, carthamoside a1, chrysophanol-9-anthrone and glu-his-leu (Figure 7C). At the same time, mRNA T10G3.2 was identified as significantly positively correlated with metabolites such as leucogenenol, beta-funaltrexamine, carthamoside a1, chrysophanol-9-anthrone, glu-his-leu, and mRNA acdh-1 was significantly positively correlated with metabolites such as 5,6-dihydro-5′-azacytidine, cloxacillin, desglucocheirotoxol and pyroglutamylisoleucine (Figure 7C). These results revealed the intrinsic correlation between characteristic mRNAs and metabolites in *C. elegans* following NL intervention, highlighting the synergistic interplay between transcriptional regulation and metabolic reprogramming [23]. This integrated multi-omics analysis provides systematic evidence for deciphering the molecular pathways that translate upstream gene expression changes into downstream metabolic phenotypes, and facilitates the identification of core effector molecules responsible for the antioxidant activity of NL [62]. Moreover, the combined transcriptomic and metabolomic approach overcame the limitations of single-omics analysis, enabling a comprehensive interpretation from regulatory gene networks to functional metabolic shifts [61]. This strategy not only deepened the mechanistic understanding of NL-mediated antioxidant effects but also provided a valuable reference for exploring the biological functions of other natural products in regulating stress responses and redox homeostasis [63]. However, the current analysis only demonstrated correlative associations between mRNAs and metabolites, without establishing definitive causal relationships [64]. Therefore, further studies are required to validate the causal links among key genes and metabolites, clarify the regulatory directionality of transcriptional control on metabolic profiles, and construct a complete causal regulatory network. In addition, targeted interventions based on the identified hub genes and metabolites could be designed to evaluate their potential roles in enhancing organismal antioxidant capacity, delaying aging, and ameliorating oxidative stress-related diseases.

## 4. Conclusions

In this study, the potential anti-OS activity of NHDC was explored through network pharmacology and molecular docking technology, revealing that NHDC could ameliorate potential OS conditions by targeting and binding to CYP19A1, TYR, EPHX2, TDP1, ESR1, and SLC5A1 proteins. Meanwhile, the enhancement of antioxidant capacity markers under non-stressed conditions of NL was investigated by elucidating its effects on the antioxidant capacity of *C. elegans*: the MDA level in *C. elegans* intervening with NL was significantly reduced to 0.65 ± 0.06 nmol/mg prot, and the activities of antioxidant enzymes T-SOD, GSH-Px, and CAT were significantly increased to 48.83 ± 1.75, 112.95 ± 0.55, and 6.30 ± 0.16 U/mg prot, respectively. In addition, transcriptomic and metabolomic technologies were applied to decipher the mechanism underlying NL-enhanced potential antioxidant capacity in *C. elegans* at the molecular and metabolite levels. Longevity regulating pathway–worm was identified as a potential key signaling pathway at the molecular level, and the pentose phosphate pathway was identified as the key metabolic pathway mediating the antioxidant capacity enhancement of *C. elegans* upon NL intervention. These results provide multi-dimensional evidence for the antioxidant activity of NHDC, ranging from in vitro target prediction to in vivo validation, and form molecular pathway analysis to metabolic mechanism elucidation, which could clarify the core regulatory logic of target–pathway–antioxidant activity, and offer systematic theoretical support for the synergistic development of NHDC and NL in the field of antioxidants, as well as their applications in the functional food and flavored food industries. Nevertheless, this study has certain limitations in that only biochemical antioxidant markers were evaluated in *C. elegans* without assessing lifespan, survival rate, or stress resistance under exogenous oxidative stress; future research will further conduct basal lifespan assays and acute oxidative stress survival experiments to verify whether the observed molecular and metabolic changes confer actual protective effects and organismal resilience.

## Figures and Tables

**Table 1:** Grouping and intervention conditions of *Caenorhabditis elegans*.

Group	Intervention Concentration	Intervention Dose
NFD	OP50	0.1 mL
NL-L	NL was dissolved in OP50 to a final concentration of 50 μg/mL	0.1 mL
NL-M	NL was dissolved in OP50 to a final concentration of 100 μg/mL	0.1 mL
NL-H	NL was dissolved in OP50 to a final concentration of 200 μg/mL	0.1 mL

**Table 2:** Key protein docking site information for NHDC regulation of OS diseases.

Number	Protein Name	Vina Score (kcal/mol)	Conventional Hydrogen Bonds	C-H Hydrogen Bonds
A	CYP19A1	−9.8	ALAA306, THRA310, ARGA145, GLYA436, ARGA435	THRA310, SERA314, ILEA133
B	TYR	−9.5	THRA240, GLYA239, GLYA237, ARGA6, TYRB68, SERB53, TYRA27	TYRB64
C	EPHX2	−9.0	PROA227, THRA230, THRA532	HISA146
D	TDP1	−8.8	SERA459, ASNA283, GLUA538, SERA536, SERA463, SERA400	VALA401
E	ESR1	−8.8	LEUB346, GLUB353, THRB347	GLYB521
F	SLC5A1	−8.8	THRA362, ALAA93, GLNA451, ASPA424, ASPA273, SERA449, CYSA511	ALAA344, SERA449, GLYA509

## Data Availability

The original contributions presented in this study are included in the article. Further inquiries can be directed to the corresponding author.

## References

[1] Genitsaridi I., Salpea P., Salim A., Sajjadi S.F., Tomic D., James S., Thirunavukkarasu S., Issaka A., Chen L., Basit A. (2026). 11th edition of the IDF Diabetes Atlas: Global, regional, and national diabetes prevalence estimates for 2024 and projections for 2050. Lancet Diabetes Endocrinol..

[2] He F., Liu J., Huang Y., Chen L., Rizi E.P., Zhang K., Ke L., Loh T.P., Niu M., Peng W.K. (2024). Nutritional load in post-prandial oxidative stress and the pathogeneses of diabetes mellitus. npj Sci. Food.

[3] Benucci I., Lombardelli C., Esti M. (2025). A comprehensive review on natural sweeteners: Impact on sensory properties, food structure, and new frontiers for their application. Crit. Rev. Food Sci..

[4] Dragomir N., Grigore D., Pogurschi E.N. (2025). Beyond sugar: A holistic review of sweeteners and their role in modern nutrition. Foods.

[5] Choi S., Yu S., Lee J., Kim W. (2021). Effects of neohesperidin dihydrochalcone (NHDC) on oxidative phosphorylation, cytokine production, and lipid deposition. Foods.

[6] Yang R., Qi L., Liang W. (2023). Neohesperidin dihydrochalbazone protects against septic acute kidney injury in mice. Phytomedicine.

[7] Kwon M., Kim Y., Lee J., Manthey J.A., Kim Y., Kim Y. (2022). Neohesperidin dihydrochalcone and neohesperidin dihydrochalcone-O-glycoside attenuate subcutaneous fat and lipid accumulation by regulating PI3K/AKT/mTOR pathway In Vivo and In Vitro. Nutrients.

[8] Shah R., de Jager L.S. (2017). Recent analytical methods for the analysis of sweeteners in food: A regulatory perspective. Food Drug Adm. Pap..

[9] Luiza Koop B., Nascimento Da Silva M., Diniz Da Silva F., Thayres Dos Santos Lima K., Santos Soares L., José De Andrade C., Ayala Valencia G., Rodrigues Monteiro A. (2022). Flavonoids, anthocyanins, betalains, curcumin, and carotenoids: Sources, classification and enhanced stabilization by encapsulation and adsorption. Food Res. Int..

[10] Katelia P., Saini R., Dhiman A. (2025). Micronization by supercritical fluid technologies for green encapsulation and stabilization of food bioactives: A comprehensive review of mechanisms and applications. Food Biosci..

[11] Sancineto L., Ostacolo C., Ortega-Alarcon D., Jimenez-Alesanco A., Ceballos-Laita L., Vega S., Abian O., Velazquez-Campoy A., Moretti S., Dabrowska A. (2021). L-arginine improves solubility and anti SARS-CoV-2 Mpro activity of rutin but not the antiviral activity in cells. Molecules.

[12] Mancillas-Quiroz J.A., Carrasco-Portugal M.D.C., Mondragón-Vásquez K., Huerta-Cruz J.C., Rodríguez-Silverio J., Rodríguez-Vera L., Reyes-García J.G., Flores-Murrieta F.J., Domínguez-Chávez J.G., Rocha-González H.I. (2024). Development of a novel co-amorphous curcumin and l-arginine (1:2): Structural characterization, biological activity and pharmacokinetics. Pharmaceutics.

[13] Chowdhury M.A.K. (2012). Assessing Quality of Novel Plant Proteins for Salmonids. Ph.D. Thesis.

[14] Vaja R., Lopes-Pires M., Shala F., Cypaite N., Vinokurova M., Ferreira P., Mitchell J.A., Kirkby N.S. (2024). L-arginine supplementation protects against thrombosis and renal dysfunction in mice treated with the cyclooxygenase-2 inhibitor parecoxib. J. Thromb. Haemost..

[15] Lin S., Wang Y., Huang C., Niu Y., Liu Y., Xiao W., Chen F., Xue R. (2026). Co-amorphous salts of luteolin-arginine/lysine with enhanced solubility. Mater. Chem. Phys..

[16] Lee Q., Xu L., Zhou W., Yu M., Zeng D., Zhu S. (2025). Non-thermal preparation, component identification, and in vivo antioxidant activity mechanism exploration of polyphenols and derivatives from *Ananas comosus* (L.) flesh. Food Res. Int..

[17] Li N., Li Q., He X., Gao X., Wu L., Xiao M., Cai W., Liu B., Zeng F. (2022). Antioxidant and anti-aging activities of *Laminaria japonica* polysaccharide in *Caenorhabditis elegans* based on metabonomic analysis. Int. J. Biol. Macromol..

[18] Duan H., Yu Q., Ni Y., Li J., Yu L., Yan X., Fan L. (2024). Dose-dependent effect of *Dendrobium officinale* polysaccharide on anti-aging in *Caenorhabditis elegans*: A metabolomics analysis focused on lipid and nucleotide metabolism regulation. Food Biosci..

[19] Dong Y., Chen C., Wang P., Wang C., Fu X. (2026). Sugarcane extract enhances stress resilience and longevity in *Caenorhabditis elegans*: NMR structural elucidation and transcriptomic mechanisms. Food Res. Int..

[20] Lee Q., Xu L., Zhu S. (2026). Non-thermal continuous preparation of pineapple (*Ananas comosus* (L.)) flavored fruit powder: Identification of characteristic volatile flavor components and exploration of in vivo antioxidant activity mechanism. Food Chem..

[21] Wen X., Li X., Zhu S., Chen L., Wang Z., Yu M., Deng Z. (2025). Interaction mechanism and structure-activity relationship of hesperidin, methyl hesperidin and hesperidin methyl chalcone with ovalbumin: A comparative study. Food Chem..

[22] Lee Q., Xu L., Zhou W., Zhu S. (2025). High voltage pulsed electric field combined with ultrasound as a green processing technology to enhance the flavor and nutritional characteristics of pineapple pulp dissolved substances. Food Wellness.

[23] Liu Y., Wang Y., Liang Y., Yang S., Deng Y., Zeng S., Wang Y., Shu Z., Shuai Y., Guo H. (2025). Transcriptomics and metabolomics revealed the effects of *Polygonatum Rhizoma* polysaccharide on delaying *C. elegans* senescence and ameliorating Alzheimer’s disease. Int. J. Biol. Macromol..

[24] Lee Q., Xue Z., Zheng M., Liu B., Zeng F. (2026). Antioxidant activity of low molecular weight polysaccharides from *Tremella fuciformis* in *Caenorhabditis elegans* based on metabolomics analysis. J. Future Foods.

[25] Liu X., Wang C., Tang S., Wang G., Huang Y., Yang F., Tan X., Bai J., Huang L. (2025). Comparative study on the alleviating effect of neohesperidin dihydrochalcones and its synthetic precursor neohesperidin on ovalbumin-induced food allergy. Food Res. Int..

[26] Wang P., Tao F., Dai Z., Wang T., Zhang C., Fan H., Qin M., Qi C., Li Y., Hao J. (2024). Neohesperidin dihydrochalcone can improve intestinal structure and microflora composition of diabetic zebrafish. J. Funct. Foods.

[27] Chen L., Ma N., Liu D., Li Y., Ci X., Wei Z. (2025). Tiliroside induces ferroptosis and suppresses tumor growth by synergistically targeting AKR1B1 and modulating iron metabolism in ovarian cancer cells. Eur. J. Pharmacol..

[28] Thakur P., Mittal N., Chaudhary J., Kamboj S., Jain A. (2025). Unveiling the substantial role of rutin in the management of drug-induced nephropathy using network pharmacology and molecular docking. Int. Immunopharmacol..

[29] P M., Thirumurugan A., Achiraman S., Senthil Kumar T. (2025). Protective effect of hydroalcoholic *Crateva religiosa* G. Forst. bark extract on oxidative stress, hormonal imbalance and gene expressions (CYP19A1 and PPARγ) in letrozole-induced polycystic ovarian syndrome rats. J. Ethnopharmacol..

[30] Yao Y., Shao L., Li Z., Liang J., Zhu C., Wang S., Lin C., Liu F. (2026). Xiebai San mediates the NCOA2-ESR1/2 signaling pathway to intervene in interstitial pneumonia. Phytomedicine.

[31] Zhou M., Ma J., Kang M., Tang W., Xia S., Yin J., Yin Y. (2024). Flavonoids, gut microbiota, and host lipid metabolism. Eng. Life Sci..

[32] Passero P., Muthular M., Barceló S., Miozza V., Pérez C. (2022). Inhibition of azole-resistant *Candida albicans* ATPase and oxidoreductase activity by a flavonoid from *Dalea elegans*. J. Med. Mycol..

[33] Guo M., Zhao H., Song N., Huang P., Li M., Han L., Zeng K., Lu Z. (2025). Shenmai injection attenuates sepsis-associated acute lung injury by remodeling gut microbiota and restoring steroid hormone biosynthesis. Fitoterapia.

[34] Zhao D., Yang S., Cai M., Bian Y., Qiu Y., Yao Q. (2026). Enzyme pretreatment combined with ultrasound-assisted aqueous biphasic extraction of flavonoids from *Abelmoschus manihot* (L.) leaves: Process optimization, antioxidant activity, and molecular docking. Food Chem..

[35] Li X., Luo Z., Hu T., Ou D., Guo R., Li C., Yang J., Song X. (2025). Exploring the anti-inflammatory effects of total flavonoids from *L. gracile* on LPS-induced inflammation: An integrated approach combining network pharmacology, molecular docking, and experimental validation. Int. Immunopharmacol..

[36] Tian F., Sun S., Ge Z., Ge Y., Ge X., Shi Z., Qian X. (2025). Understanding the anticancer effects of phytochemicals: From molecular docking to anticarcinogenic signaling. J. Nutr..

[37] Tsikas D. (2017). Assessment of lipid peroxidation by measuring malondialdehyde (MDA) and relatives in biological samples: Analytical and biological challenges. Anal. Biochem..

[38] Yekti R., Bukhari A., Jafar N., Thaha A.R. (2018). Measurement of malondialdehyde (MDA) as a good indicator of lipid peroxidation. Int. J. Allied Med. Sci. Clin. Res..

[39] Zhang S., Cao Z., Yin T., Yi C., He H., Huo J., Yuan J. (2025). Antioxidant effects of whole-grain rice and products: Multiple perspectives of chemistry, cell, and *Caenorhabditis elegans*. J. Future Foods.

[40] Daly K., Darby A.C., Shirazi-Beechey S.P. (2016). Low calorie sweeteners and gut microbiota. Physiol. Behav..

[41] Sun Y., Liang J., Zhang Z., Sun D., Li H., Chen L. (2024). Extraction, physicochemical properties, bioactivities and application of natural sweeteners: A review. Food Chem..

[42] Lee Q., Xue Z., Luo Y., Lin Y., Lai M., Xu H., Liu B., Zheng M., Lv F., Zeng F. (2024). Low molecular weight polysaccharide of *Tremella fuciformis* exhibits stronger antioxidant and immunomodulatory activities than high molecular weight polysaccharide. Int. J. Biol. Macromol..

[43] Miao Y., Xu Y., Gao J., Ai X., Duan R., Li R. (2025). Transcriptomic analysis reveals molecular mechanism by which Chinese olive fruit prolongs lifespan of *Caenorhabditis elegans*. npj Sci. Food.

[44] Lei J., Cao L., Li Y., Kan Q., Yang L., Dai W., Liu G., Fu J., Chen Y., Huang Q. (2024). Physiological evaluation and transcriptomic and proteomic analyses to reveal the anti-aging and reproduction-promoting mechanisms of glycitein in *Caenorhabditis elegans*. Food Funct..

[45] Drobny A., Meloh H., Wächtershäuser E., Hellmann B., Mueller A.S., van der Klis J.D., Fitzenberger E., Wenzel U. (2020). Betaine-rich sugar beet molasses protects from homocysteine-induced reduction of survival in *Caenorhabditis elegans*. Eur. J. Nutr..

[46] Zhang X., Meng X., Liu Y., Yang X., Chen J., Liu T., Liao Z., Fang X., Wang J. (2025). Roles of oolong tea extracts in the protection against Staphylococcus aureus infection in *Caenorhabditis elegans*. J. Food Sci..

[47] Lee K., Goh G.Y.S., Wong M.A., Klassen T.L., Taubert S. (2016). Gain-of-function alleles in *Caenorhabditis elegans* nuclear hormone receptor nhr-49 are functionally distinct. PLoS ONE.

[48] Evans E.A., Chen W.C., Tan M.W. (2008). The DAF-2 insulin-like signaling pathway independently regulates aging and immunity in *C. elegans*. Aging Cell.

[49] Wang S., Lin D., Cao J., Wang L. (2023). APPA increases lifespan and stress resistance via lipid metabolism and insulin/IGF-1 signal pathway in *Caenorhabditis elegans*. Int. J. Mol. Sci..

[50] Shrivastava A., Sandhof C.A., Reinle K., Jawed A., Ruger-Herreros C., Schwarz D., Creamer D., Nussbaum-Krammer C., Mogk A., Bukau B. (2022). The cytoprotective sequestration activity of small heat shock proteins is evolutionarily conserved. J. Cell Biol..

[51] Hu R., Zhang Y., Qian W., Leng Y., Long Y., Liu X., Li J., Wan X., Wei X. (2022). *Pediococcus acidilactici* promotes the longevity of *C. elegans* by regulating the insulin/IGF-1 and JNK/MAPK signaling, fat accumulation and chloride ion. Front. Nutr..

[52] Xie J., Cao Z., Chen X., Xie J., Chen Y., Hu X., Sun N., Song Y., Wu Y., Yu Q. (2026). Integrating network pharmacology and transcriptomics reveals that bound polyphenols from defatted rice bran insoluble dietary fiber delays senescence in C. elegans through modulating IIS pathway. Food Biosci..

[53] Tao M., Huang Y., Xu T., Peng X., Liao X., Xia Z., Zheng D., Li R., Xu X. (2025). Anti-infective properties of mung bean (*Vigna radiata* (L.)R. Wilczek) coat extract on Pseudomonas aeruginosa-infected *Caenorhabditis elegans*: Transcriptomics and pathway analysis. J. Ethnopharmacol..

[54] Thongdechsri S., Kamonnat C., Sanguanphun T., Kraokaew P., Jattujan P., Niamnont N., Smith S.J., Cummins S.F., Jongkamonwiwat N., Sobhon P. (2026). Triterpenoid saponin-containing Holothuria leucospilota extract mitigates amyloid-β proteotoxicity in transgenic C. elegans through activations of protein clearance and stress resistance pathways. Food Biosci..

[55] Shen W., Yuh C., Lu Y., Lin Y., Ching T., Wang C., Wang H. (2023). Reduced ribose-5-phosphate isomerase A-1 expression in specific neurons and time points promotes longevity in *Caenorhabditis elegans*. Antioxidants.

[56] Bradshaw P.C. (2019). Cytoplasmic and mitochondrial NADPH-coupled redox systems in the regulation of aging. Nutrients.

[57] Gao Y., Li C., Li J., Duan M., Li X., Zhao L., Wu Y., Gu S. (2024). *Weizmannia coagulans* BC99 alleviates hyperuricemia and oxidative stress via DAF-16/SKN-1 activation in *Caenorhabditis elegans*. Front. Microbiol..

[58] Seregina T.A., Shakulov R.S., Petrushanko I.Y., Lobanov K.V., Mironov A.S. (2025). Biosynthesis of ribose-5-phosphate—Metabolic regulator of *Escherichia coli* viability. Cells.

[59] Li Y., Wang R., A J., Sun R., Na S., Liu T., Ding X., Ge W. (2022). Cerebrospinal fluid metabolic profiling reveals divergent modulation of pentose phosphate pathway by midazolam, propofol and dexmedetomidine in patients with subarachnoid hemorrhage: A cohort study. Bmc Anesthesiol..

[60] Riganti C., Gazzano E., Polimeni M., Aldieri E., Ghigo D. (2012). The pentose phosphate pathway: An antioxidant defense and a crossroad in tumor cell fate. Free Radic. Bio. Med..

[61] Li Q., Xiao M., Li N., Cai W., Zhao C., Liu B., Zeng F. (2023). Application of *Caenorhabditis elegans* in the evaluation of food nutrition: A review. eFood.

[62] Ji J., Kang X., Wang B., Yuan H., Jiang Z. (2026). The mechanism of oxidative stress induced by nanoplastics in Caenorhabditis elegans: Integrated analysis of transcriptomics and metabolomics. Comp. Biochem. Physiol. Part C Toxicol. Pharmacol..

[63] Shi Q., Chen H., Wu Y., Tong P., Gao J. (2023). Integrative analysis of transcriptomic and metabolomic profiles uncovers health-promoting mechanisms of ovotransferrin in Caenorhabditis elegans. J. Funct. Foods.

[64] Zheng H., Su C., Wang Z., Zhao L., Li W. (2025). Review on potential of Caenorhabditis Elegans as an anti-aging evaluation model for polysaccharides. Int. J. Biol. Macromol..

